# Gastrointestinal tract metastasis of mandibular diffuse large B‐cell lymphoma

**DOI:** 10.1002/ccr3.1704

**Published:** 2018-07-10

**Authors:** Turker Yucesoy, Erdem Kilic, Hakan Ocak, Alper Alkan, Kemal Deniz

**Affiliations:** ^1^ Faculty of Dentistry Department of Oral and Maxillofacial Surgery Bezmialem Vakif University Istanbul Turkey; ^2^ Department of Oral and Maxillofacial Surgery Private Clinic Kayseri Turkey; ^3^ Department of Pathology, Medicine Faculty Erciyes University Kayseri Turkey

**Keywords:** critical care medicine, dentistry, gastroenterology and hepatology, oncology

## Abstract

Not only diffuse large B‐cell lymphoma is a malignancy, but also is initially and orally diagnosed in early stages. However, it could be misdiagnosed with other oral pathologies. However yet, early diagnosis is still crucial for the prognosis, morbidity, and mortality in such cases. Additionally, whole‐body scanning with positron emission tomography/computed tomography should be performed for diagnosis and treatment process.

## 1 INTRODUCTION

Diffuse large B‐cell lymphoma (DLBCL) is generally seen extra‐nodally or in salivary glands and gastrointestinal tract. Early diagnosis is crucial for the prognosis, morbidity, and mortality. Here, we report a patient with DLBCL presenting as the first sign of oral localization and its management and emphasize the importance of diagnostic biopsy.

Hodgkin's lymphoma (HL) develops as a nodal disease whereas Non‐Hodgkin's Lymphoma (NHL) may be evolved extranodally. Salivary glands and gastrointestinal tract are another anatomic areas where NHL is seen. It is very unusual that this lesion will be seen orally and elsewhere in the body simultaneously.^1^, ^2^ NHL's are rarely detected primarily in the bone and bone location could be both maxilla and mandible,^3^ however, they can be found in both the maxilla and mandible with a slight predilection to the maxilla.^4^

Diffuse large B‐cell lymphoma is the most common type of NHLs. It presents as a fast‐growing lesion/tumor, characteristically in lymph nodes, spleen, liver, bone marrow and rarely, in other organs.^5^ 30%‐40% of these cases are involved in the head and neck region.^6^ DLBCL is the largest subtype group of NHL with the incidence of 2.9/100.000 per year and according to WHO Classification.^7^

Primary malignant NHLs of mandible represent about 0.8% of all the tumors in this bone and about 0.6% of all malignant NHLs.^8^ DLBCL is occasionally encountered in the body of the mandible and thus it may present a diagnostic challenge. Hodgkin's disease is classically characterized by multinucleate Ree—Sternberg cells. All other neoplasms of the lymphoid system are referred to NHL and are derived predominantly from the cells of B‐lymphocyte series.^9^, ^10^ DLBCL is histologically seen as diffuse sheets of large cells with vesicular nuclei, prominent nucleoli, basophilic cytoplasm and a moderate to high proliferation fraction with positive immunohistochemistry for B cell‐associated antigens (CD19, CD20, CD22, CD79a).^11^

Additionally, it is indicated to take a bone scan, after detection of clinical signs of osseous involvement through the mandible CT.^12^ Whole‐body bone scans and positron emission tomography/computed tomography (PET/CT) should be preferred for detection of the appendicular bone involvement of DLBCL.^13^

## 2 CASE REPORT

### 2.1 Presenting complications

A 72‐year‐old Caucasian man was admitted to our department with a pain in the left posterior mandible and periodontal hyperplasia associated with the left mandibular second molar tooth. He also complained about the ongoing pain for 2 months and spontaneous hemorrhage within the lesion region.

### 2.2 Past history

According to his medical history, the patient had suffered from coronary angioplasty 6 years ago. He also suffered from malaise and fatigue for last 1 year and also inappetence for last 6 months.

### 2.3 Clinical examination findings

Extraoral examination of the patient showed no visible swelling, tenderness or pus discharge. Skin color and temperature were normal. In the intraoral examination of the relevant region, oral hygiene level was not good and gingival tissue around the second molar was hyperplastic and had a tendency to spontaneous bleeding.

### 2.4 Radiographic report

**Figure 1:**
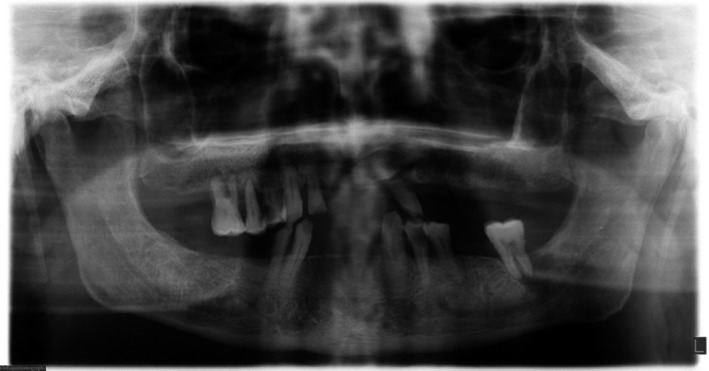
Diagnostic Panoramic Radiography Shows Border Irregularity and Radiolucency in Apical Area of a Second Left Molar in the Mandible

On the other hand, the panoramic radiograph (PANO) showed a radiolucent lesion with irregular margins located the periapical area and also extended coronally that led into the serious mobility of mandibular left second molar tooth (Figure 1).

### 2.5 Biopsy procedures

**Figure 2:**
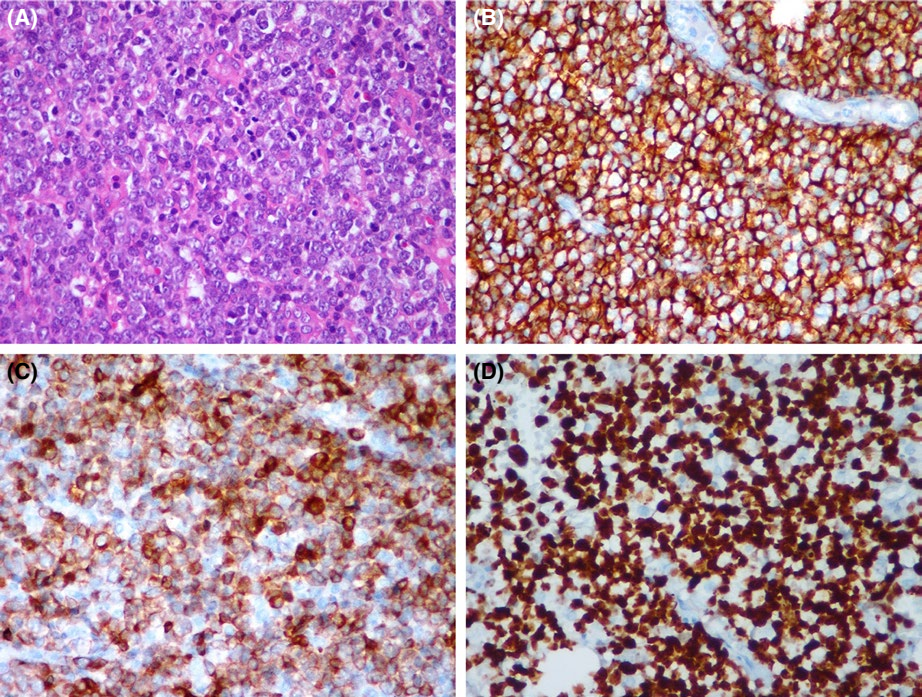
A, Light Microscopic Image from the Oral Mucosa Showing Diffuse Infiltration of Atypical Lymphoid Cells (Hematoxylin and Eosin, ×200). B, Atypical Lymphoid Cells were Positive for CD20 (×200) and (C) bcl‐2 (×200) with (D) High Proliferation Index (Ki67 = %90; (×200)

Considering the patient's medical history and after an exhaustively clinical and radiographical examination, we decided to perform an incisional biopsy under local anesthesia. As expected, the result of the pathological examination was peripheral giant cell granuloma (PGCG). We did not consider to take an initial photography before the surgical procedures, however, with regard to the pathological results, we decided to perform another surgery under local anesthesia 1 week later, including extraction of the tooth and a wide curettage of the lesion in the left posterior mandible. Despite the anesthetic procedures were performed properly and adequately, the patient was still suffering from pain but no severe hemorrhage during the curettage was observed from the surgical area. After pathological assessment of the second biopsy, the lesion was diagnosed as DLBCL (Figure 2).

### 2.6 Patient management after diagnosis of DLBCL

Even though the patient was relieved and healing was uneventful, we did several consultations and asked for PET/CT scan of entire body because of metastatic nature of DLBCL. After all of these scanning procedures, we doubted the patient may also have had DLBCL in his thyroid gland, gastric system, and prostate either.

On the other hand, 2 weeks later from the second biopsy, our patient had satisfactory outcomes, such as; no pain, hemorrhage, or swelling in the operated area. He stated that he is totally relieved and he is ready for the prosthetic procedures.

### 2.7 Pathological assessments

**Figure 3:**
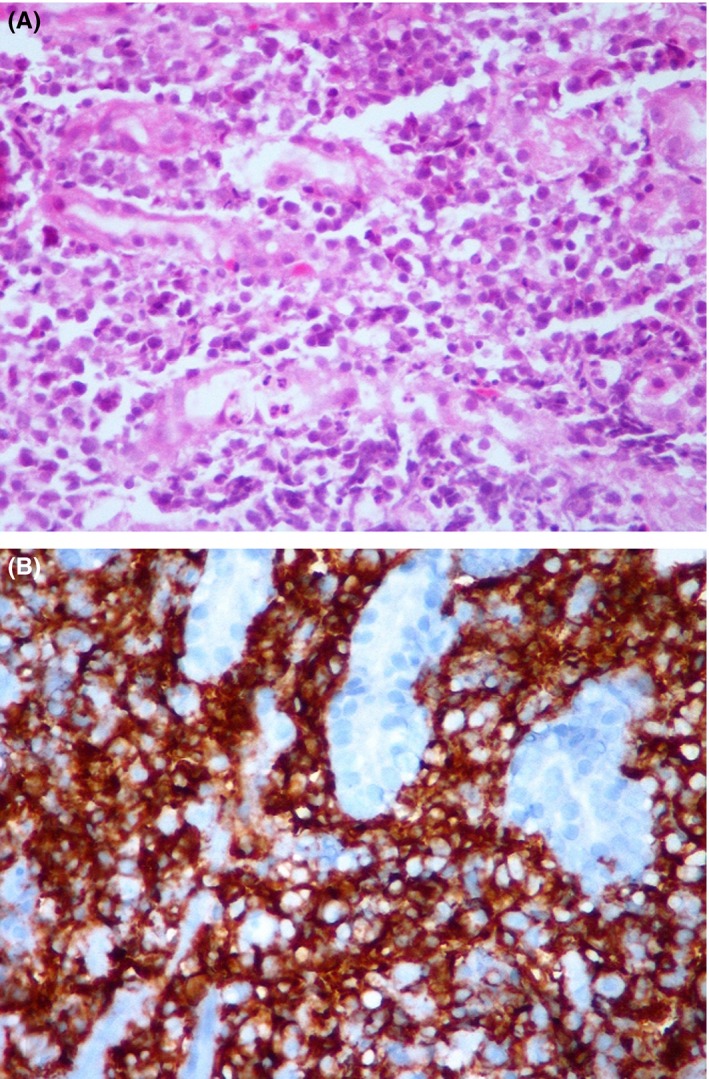
A, Photomicrograph Showing Atypical Lymphoid Cells Infiltrating the Gastric Mucosa (Hematoxylin and Eosin, ×200), (B) Which are Positive for CD20 (×200)

Mandibular biopsy results showed sheets of medium to large lymphoid cells with hyperchromatic nuclei and scanty cytoplasm. These cells stained positive for CD3, CD20, LCA1, and LCA2 for Cyclin D1 (clone Polyclonal). Ki67 proliferation index was %90. The tumor was also positive for MUM‐1 and BCL 2 and BCL 6 (Figure 2). Other blood tests and bone marrow investigations did not reveal any abnormality. However, the gastrointestinal tract biopsy resulted in almost the same outcome, with a slight difference of %80 Ki67 proliferation index, positive CD5 (Clone 4C7), and CD138 (clone MI‐15) and negative Cyclin D1 results. Attributed to these results, the lesion in the gastrointestinal tract was diagnosed with DLBCL (Figure 3).

### 2.8 Prognosis of the case

**Figure 4:**
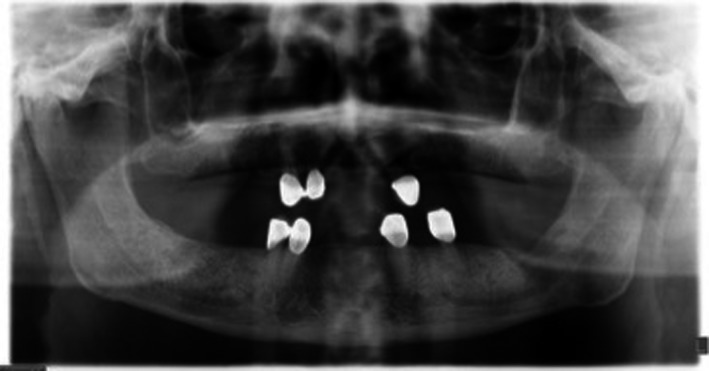
Panoramic Radiography was taken after 3 mo and Shows no Calcification in the Lesion

**Figure 5:**
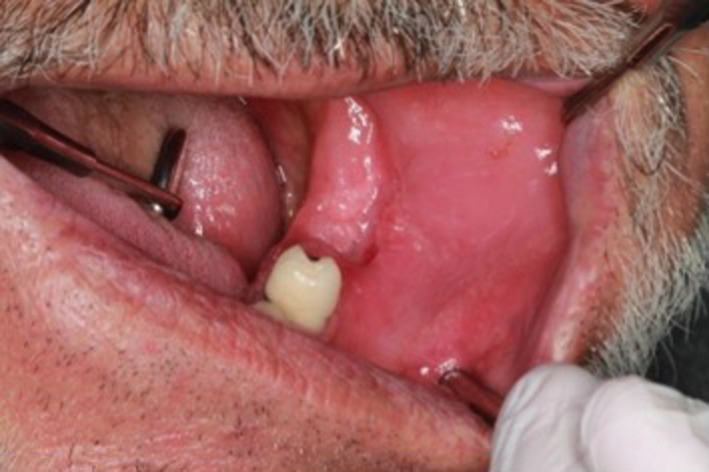
Intraoral appearance reveals that periodontal healing is uneventful

Even though radiological findings were not satisfactory enough in our second and the third follow‐up (Figure 4) and intraoral healing of the patient was still uneventful after 3 months (Figure 5), the patient was decided to have several courses of systemic chemotherapy by the department of oncology as further treatment protocol of DLBCL.

Nevertheless, the patient had a stroke after chemotherapy finished (6 months later) and he passed away within 2 years after diagnosis due to DLBCL.

## 3 DISCUSSION

Diffuse large B‐cell lymphoma is the most common type of NHL rating 30%‐40% of all cases.^14^ As one of the greatest portion of all NHLs occurring in extranodal sites (40%), the gastrointestinal tract is the most common site. Another common site for extranodal NHLs is head and neck area with Waldeyer's ring involvement.^15^ The average age of lymphoma is between 50‐55 years. Male predominance with the ratio of 3:2 is also presented.^1^ Even though this high incidence of both parts of the body, it is strange not to encounter a DLBCL case in literature, either mandible or gastrointestinal tract simultaneously, until present case.

Swelling in mandible with or without pain is one of the nonspecific and frequently observed symptoms of extranodal lymphomas in mandible where neuropathic disorders, loose teeth, and cervical adenopathy are less common.^16^ Our case had pain and spontaneous hemorrhagia in the left posterior part of mandible previously. And after wide curettage, paresthesia on the left side is observed 1 week later. On the other hand, except that complication, after 6 months the healing was uneventful and the patient was completely relieved of the pain and swelling symptoms. Because most of the lesions are associated with teeth and occasionally in the posterior part of the mandible, the definitive diagnosis could be very difficult in the maxillofacial area according to only radiographical and clinical examination.

B‐cell included concurrent granulomas of the NHLs are NOS; “not otherwise specified”, FCCL: “low‐grade lymphoma, follicular center cell lymphoma”, SLL; “small lymphocytic lymphoma”, NOS [LCL, NOS]: “large cell lymphoma”, and other types of T‐cell NHL.^17^ The phenomenon of granulomas in Hodgkin lymphoma or small non‐cleaved cell lymphoma confers a favorable prognosis.^18^, ^19^ Nyunt et al^20^ have reported a case of DLBCL associated with chronic granulomatous inflammation, which was previously misdiagnosed as tuberculosis and treated with anti‐tuberculous drugs. We also had a similar problem in our case that made us confused about surgical protocols. In our case, the first result of biopsy specimen was peripheral giant cell granuloma and the second result was DLBCL. Despite the extensive local destruction, systemic manifestations of malignant disease, such as weight loss, fever, and malaise, are very minimal.^21^ However, our patient had malaise, weight loss and inappetence even thought the destruction was not so extensive conversely to literature.

LBCL development is about 30%‐40% of adult NHLs whose median age is the sixth decade. However, a wide range of age is likely as lesions may be seen in children either. LCLs’ nature is aggressive but fortunately curable with aggressive therapy.^22^ Currently, the standard of care includes systemic chemotherapy which leads to a cure in most of the patients.^23^, ^24^ On the other hand, the combination of radio/chemotherapy strategy seems promising in recent studies but more exhaustively evaluation is required in primary bone DLBCL.^25^ As recommended, the patient received several courses of chemotherapy and no radiotherapy was needed.

The follicular, small cleaved‐cell type are considered low grade with a 5‐year survival rate of 70%, however, DLC types of NHL are considered high grade and 5‐year survival of DLBCL is about 30%, as our patient passed away in the second year of diagnosis.^26^ Some authors reported that the pathological evaluations revealed positive for CD20 and the high percentage of Ki67 for diagnosis of DLBCL.^27^, ^28^

It is challenging to detect primary DLBCL when it affects more than one organ of a human body. Even though it is rarely seen in the oral region, clinicians should keep in mind that differential diagnosis of these lesions is very difficult when other lesions are involved or related. However, early diagnosis is still crucial for the diagnosis, prognosis, morbidity, and mortality in such metastasis cases. Scanning whole‐body with PET/CT is highly recommended for diagnosis and treatment process.

## CONFLICT OF INTEREST

None declared.

## AUTHORSHIP

TY: Wrote the main part of the manuscript according to collected data, performed the surgical procedures and followed up with the patient. EK: Helped to data interpretation and manuscript evaluation, also helped to follow up with the patient. HO: Helped to evaluate and edit the manuscript. AA: Supervised development of work, helped in data interpretation and manuscript evaluation. KD: Performed histopathological evaluations and wrote “Pathological Assessments” part of the manuscript.
